# Clinicopathologic and Prognostic Value of Serum Carbohydrate Antigen 19-9 in Gastric Cancer: A Meta-Analysis

**DOI:** 10.1155/2015/549843

**Published:** 2015-10-21

**Authors:** Yong-xi Song, Xuan-zhang Huang, Peng Gao, Jing-xu Sun, Xiao-wan Chen, Yu-chong Yang, Cong Zhang, Hong-peng Liu, Hong-chi Wang, Zhen-ning Wang

**Affiliations:** Department of Surgical Oncology and General Surgery, First Hospital of China Medical University, 155 North Nanjing Street, Heping District, Shenyang 110001, China

## Abstract

*Background.* The clinical value of carbohydrate antigen (CA) 19-9 in gastric cancer is controversial. We evaluated the clinicopathologic and prognostic value of CA 19-9 in gastric cancer.* Methods.* A literature search was conducted in PubMed and Embase databases. Odds ratios (ORs), risk ratios (RR), hazard ratios (HRs), and 95% confidence intervals (CIs) were used as effect measures.* Results*. Thirty-eight studies were included. Results showed that there were significant differences in the incidence of high CA 19-9 levels between stages III/IV and I/II groups (OR = 3.36; 95% CI = 2.34–4.84), the pT3/T4 and pT1/T2 groups (OR = 2.40; 95% CI = 1.60–3.59), the lymph node-positive and node-negative groups (OR = 2.91; 95% CI = 2.21–3.84), the metastasis-positive and metastasis-negative groups (OR = 2.76; 95% CI = 1.12–6.82), and vessel invasion-positive and invasion-negative groups (OR = 1.66; 95% CI = 1.11–2.48). Moreover, CA 19-9 was significantly associated with poor overall survival (HR = 1.83; 95% CI = 1.56–2.15), disease-free survival (HR = 1.85; 95% CI = 1.16–2.95), and disease-specific survival (HR = 1.33; 95% CI = 1.10–1.60) in gastric cancer.* Conclusions*. Our meta-analysis showed that CA 19-9 indicates clinicopathologic characteristics of gastric cancer and is associated with a poor prognosis.

## 1. Introduction

Gastric cancer remains the fourth most commonly diagnosed cancer and third leading cause of cancer deaths worldwide [1]. Gastric cancer is often diagnosed at an advanced stage and the survival rate is low [2]. The tumor-node-metastasis (TNM) classification is the most important prognostic factor in gastric cancer, but it is still difficult to obtain complete prognostic information [3]. Therefore, it is important to identify other markers, which should be simple, feasible, and less costly, for the assessment of clinicopathologic characteristics and prediction of prognosis.

As a type of tumor-associated antigen for gastrointestinal cancer, carbohydrate antigen (CA) 19-9 is a sialylated derivative of Lewis^a^ blood group antigen [4] and is not applied to TNM staging of gastric cancer according to the American Joint Committee on Cancer (7th edition) [5]. Most research has focused on pancreatic cancer [6–8]. Therefore, the clinical value of CA 19-9 in gastric cancer remains controversial and has not been confirmed [9–11].

The aim of this meta-analysis was to evaluate the relationship between CA 19-9 and clinicopathologic characteristics and the prognostic value of CA 19-9 in gastric cancer.

## 2. Materials and Methods

### 2.1. Literature Search

A systematic literature search was conducted for relevant studies using PubMed and Embase databases. The search strategy included the following terms: “carbohydrate antigen 19-9,” “carbohydrate antigen 199,” “CA 19-9,” “CA 199,” “gastric cancer,” and “stomach cancer.” A manual search of the reference lists of the retrieved studies and reviews was also performed to identify potential studies.

### 2.2. Eligibility Criteria

Studies were included if the following inclusion criteria were met: (1) sample for measuring CA 19-9 was obtained from serum of gastric cancer; (2) studies reported the clinicopathologic or/and prognostic values of CA 19-9; and (3) outcome measures could be extracted directly or calculated from published data indirectly. Only the most informative study was included if there were several duplicated studies based on the same patient population.

### 2.3. Data Extraction and Quality Assessment

Studies were reviewed and data were independently extracted by two reviewers (Yong-xi Song and Xuan-zhang Huang). The following data were extracted: first author; country and year of publication; sample size; patients characteristics; follow-up period; sampling time; cut-off value; and tumor clinicopathologic characteristics and prognostic value (overall survival (OS), disease-free survival (DFS), and disease-specific survival (DSS)). The quality of the included studies was assessed by Newcastle-Ottawa Scale (NOS) criteria [12]. Any disagreements on the data extraction and quality assessment were resolved by comprehensive discussion.

### 2.4. Statistical Analysis

Odds ratios (ORs) and 95% confidence intervals (CIs) were used as a measure to evaluate the relationship between CA 19-9 and tumor clinicopathologic characteristics. For the relationship between CA 19-9 and prognosis, we used hazard ratios (HRs) and 95% CIs as effect measures. HRs and 95% CIs were extracted directly or calculated from available data using the methods designed by Tierney if the values were not reported directly [13]. For the studies that reported several results separately based on different subgroups, we pooled multiple effect values into an estimated value for further meta-analysis.

The Cochran *Q* test and *I* ^2^ statistics were used to evaluate heterogeneity, and statistically significant heterogeneity was defined as *p* < 0.10 and/or *I* ^2^ > 50% [14]. We used a random effects model if significant heterogeneity existed; otherwise, a fixed effects model was used. The sources of heterogeneity were explored by metaregression and subgroup analyses. Metaregression was only performed when the number of studies was greater than ten, considering the accuracy of the results [15, 16]. Publication bias was assessed using Begg's and Egger's tests, and trim-and-fill analysis was performed to assess the effect of publication bias [17–19].

All statistical analyses were conducted using STATA software (version 12.0; Stata Corporation, College Station, TX, USA), and a two-sided *p* value < 0.05 was considered statistically significant.

## 3. Results

### 3.1. Baseline Characteristics of Eligible Studies

**Figure 1:**
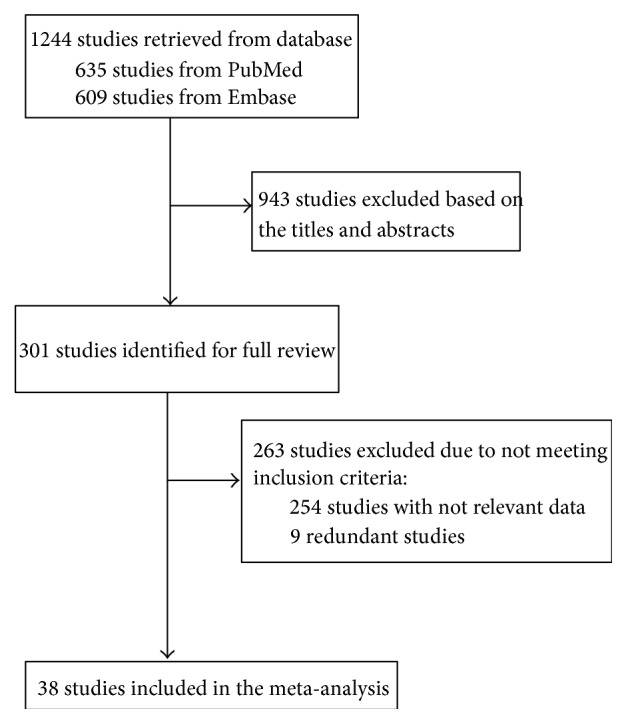
Flow diagram showing the literature search and selection process for the included studies.

A total of 1244 studies were initially identified, of which 943 were excluded after reviewing titles and abstracts. Then, 263 studies were excluded after full-text review. Finally, 38 studies were included (Figure 1) [9–11, 20–54].

The 38 eligible studies were published between 1995 and 2014 and included 11408 gastric cancer patients (mean sample size, 300; median sample size with corresponding range, 167 [23–1710]). Of the eligible studies, 30 assessed the value of preoperative CA 19-9 [9, 11, 20, 23, 25–27, 29–31, 33, 35–47, 49–54], two assessed the value of postoperative CA 19-9 [28, 34], two assessed the pre- and postoperative combined value of CA 19-9 [10, 48], and four studies did not report sampling time [21, 22, 24, 32]. HRs for OS, DFS, and DSS were available in 29 [9–11, 20–24, 26–33, 35, 36, 38, 40, 44–47, 49, 50, 52–54], seven [9, 11, 29, 34, 36, 43, 48], and six studies [25, 37, 39, 41, 42, 51], respectively. The characteristics and quality of the included studies are shown in Table 1.

### 3.2. Impact of CA 19-9 on Survival

#### 3.2.1. CA 19-9 and OS

**Figure 2:**
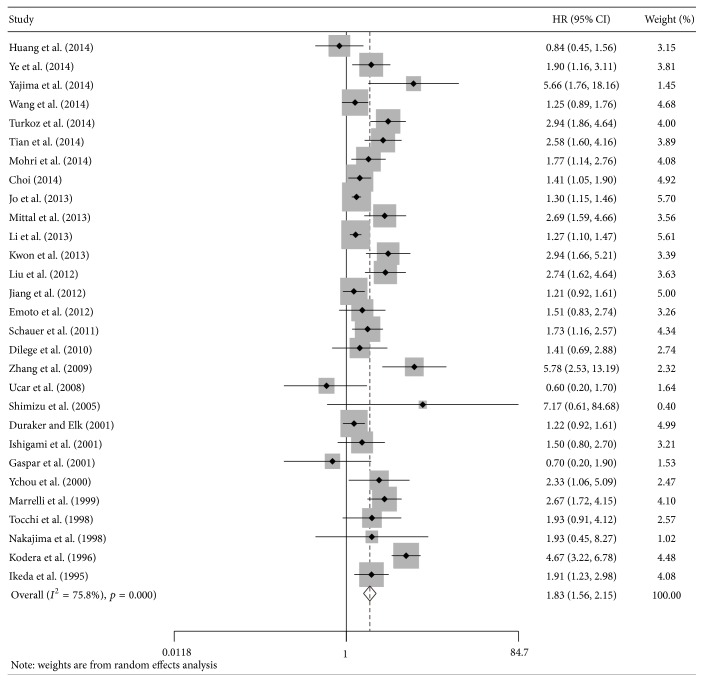
The estimated hazard ratio (HR) was summarized for the association between carbohydrate antigen 19-9 and overall survival. The left-hand column lists the first author of each study; the middle column graphically displays the effect measure for each study incorporating confidence intervals (the black solid point represents effect measure and horizontal line represents 95% confidence intervals), the area of gray square is proportional to the study's weight in the meta-analysis, and the pooled measure of effect is plotted as a diamond; the right-hand column numerically displays the effect measure, 95% confidence intervals, and weight of each study.

Twenty-nine studies evaluated the prognostic effect of CA 19-9 on OS [9–11, 20–24, 26–33, 35, 36, 38, 40, 44–47, 49, 50, 52–54]. Our results indicated that the high CA 19-9 group had a significantly shorter OS (HR = 1.83; 95%  CI = 1.56–2.15; *I* ^2^ = 75.8%; Figure 2). The result of subgroup analysis for preoperative CA 19-9 was similar (HR = 1.87; 95%  CI = 1.52–2.30; *I* ^2^ = 76.3%). As shown by the subgroup analyses stratified by cut-off value (37 U/mL and other than 37 U/mL) and study quality (NOS ≥ 6 and NOS < 6), the prognostic effect of CA 19-9 on OS was confirmed (Table 2).

#### 3.2.2. CA 19-9 and DFS

**Figure 3:**
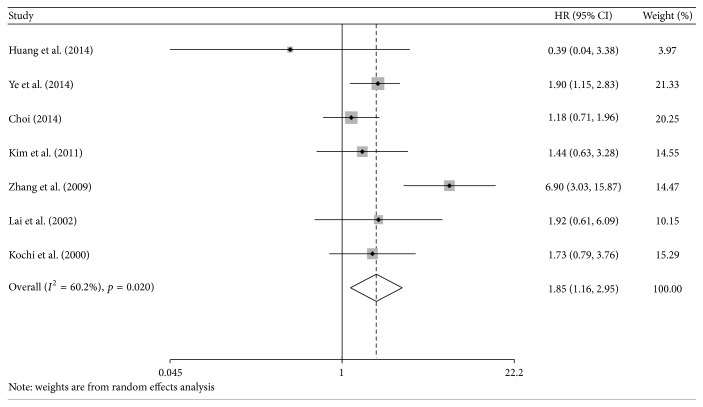
The estimated hazard ratio (HR) was summarized for the association between carbohydrate antigen 19-9 and disease-free survival. The left-hand column lists the first author of each study; the middle column graphically displays the effect measure for each study incorporating confidence intervals (the black solid point represents effect measure and horizontal line represents 95% confidence intervals), the area of gray square is proportional to the study's weight in the meta-analysis, and the pooled measure of effect is plotted as a diamond; the right-hand column numerically displays the effect measure, 95% confidence intervals, and weight of each study.

Seven studies evaluated the prognostic effect of CA 19-9 on DFS [9, 11, 29, 34, 36, 43, 48]. Our results indicated that a significantly poor DFS existed in the high CA 19-9 group (HR = 1.85; 95%  CI = 1.16–2.95; *I* ^2^ = 60.2%; Figure 3). The results for preoperative CA 19-9 levels were marginally significant (HR = 1.96; 95%  CI = 1.00–3.85; *I* ^2^ = 72.9%). Similarly, subgroup analyses for cut-off value and NOS score also showed that CA 19-9 was a significant poor prognostic factor for DFS (Table 2).

#### 3.2.3. CA 19-9 and DSS

**Figure 4:**
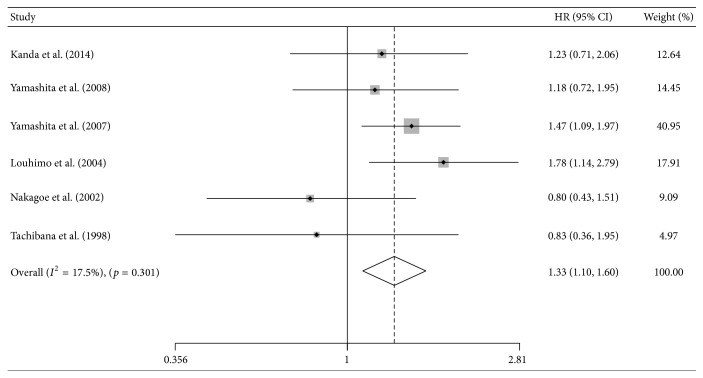
The estimated hazard ratio (HR) was summarized for the association between carbohydrate antigen 19-9 and disease-specific survival. The left-hand column lists the first author of each study; the middle column graphically displays the effect measure for each study incorporating confidence intervals (the black solid point represents effect measure and horizontal line represents 95% confidence intervals), the area of gray square is proportional to the study's weight in the meta-analysis, and the pooled measure of effect is plotted as a diamond; the right-hand column numerically displays the effect measure, 95% confidence intervals, and weight of each study.

Six studies evaluated the prognostic effect of CA 19-9 on DSS [25, 37, 39, 41, 42, 51]. Our results indicated that the high CA 19-9 group was associated with a significantly poor DSS (HR = 1.33; 95%  CI = 1.10–1.60; *I* ^2^ = 17.5%; Figure 4). Including the studies using a cut-off value of 37 U/mL, we observed a similar result (Table 2).

### 3.3. Relationship between CA 19-9 and Clinicopathologic Characteristics

#### 3.3.1. CA 19-9 and TNM Stage

**Figure 5:**
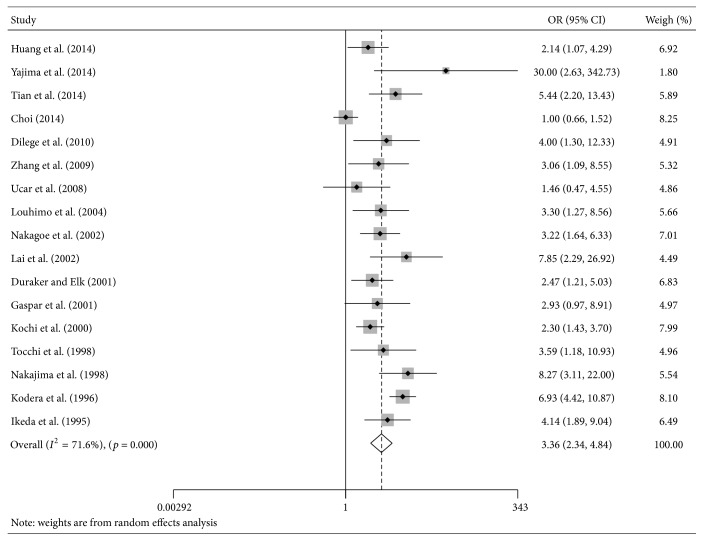
The estimated odds ratio (OR) was summarized for the association between carbohydrate antigen 19-9 and tumor stage. The left-hand column lists the first author of each study; the middle column graphically displays the effect measure for each study incorporating confidence intervals (the black solid point represents effect measure and horizontal line represents 95% confidence intervals), the area of gray square is proportional to the study's weight in the meta-analysis, and the pooled measure of effect is plotted as a diamond; the right-hand column numerically displays the effect measure, 95% confidence intervals, and weight of each study.

There were 17 studies that provided data on TNM stage and CA 19-9 [9, 20, 23, 29, 35, 36, 38, 41–43, 45, 46, 48, 50, 52–54]. Our results indicated a significantly higher incidence of high CA 19-9 levels in stages III/IV group relative to stages I/II group (OR = 3.36; 95%  CI = 2.34–4.84; *I* ^2^ = 71.6%; Figure 5). Moreover, a similar tendency was obtained in the subgroup analysis based on preoperative time, cut-off value, and study quality (Table 2).

There were significant differences in the incidence of high CA 19-9 levels between the pT3/T4 and pT1/T2 groups (OR = 2.40; 95%  CI = 1.60–3.59; *I* ^2^ = 62.8%), the lymph node-positive and lymph node-negative groups (OR = 2.91; 95%  CI = 2.21–3.84; *I* ^2^ = 55.3%), and the metastasis-positive and metastasis-negative groups (OR = 2.76; 95%  CI = 1.12–6.82; *I* ^2^ = 14.9%; Table 2).

A higher incidence of high CA 19-9 levels was observed in the peritoneal (OR = 2.20; 95%  CI = 1.25–3.90; *I* ^2^ = 85.3%) and hepatic metastasis-positive groups (OR = 3.13; 95%  CI = 1.50–6.55; *I* ^2^ = 76.9%).

#### 3.3.2. CA 19-9 and Vessel and Lymphatic Invasion

Four [21, 35, 48, 52] and three studies [48, 50, 52] assessed the relationship between CA 19-9 and vessel invasion and between CA 19-9 and lymphatic invasion, respectively. Our results showed a significantly higher incidence of high CA 19-9 levels in the vessel invasion-positive group compared with the vessel invasion-negative group, and there was no significant relationship between the CA 19-9 level and lymphatic invasion (Table 2).

#### 3.3.3. CA 19-9 and Histologic Type

Seventeen studies assessed the relationship between the CA 19-9 level and histologic type [9, 20, 21, 23, 28, 29, 35, 38, 42–46, 49, 52–54]. The results indicated that there was no significant relationship between CA 19-9 and tumor differentiation (Lauren diffuse type versus intestinal type: OR = 0.89; 95%  CI = 0.62–1.27; *I* ^2^ = 0.0%; poor differentiation versus well/moderate differentiation: OR = 0.87; 95%  CI = 0.72–1.04; *I* ^2^ = 3.4%). As shown by the subgroup analyses stratified by sampling time, cut-off value, and study quality, similar tendencies were observed.

### 3.4. Assessment of Publication Bias and Heterogeneity

The results of Begg's and Egger's tests showed no significant publication bias, except in the HRs for OS. The trim-and-fill analyses indicated that publication bias could not impact on the results for OS. Metaregression analyses showed that publication year may contribute to heterogeneity in the analyses of lymph node and hepatic metastases (lymph node: coefficient = −0.041, standard error = 0.019, and *p* = 0.046; hepatic metastasis: coefficient = −0.113, standard error = 0.021, and *p* = 0.001).

## 4. Discussion

Gastric cancer is a global health problem with a low survival rate. Tumor-associated markers are urgently needed for the diagnosis of cancer and assessment of prognosis. Nevertheless, gastric cancer-specific markers have not yet been established, and the unsatisfactory specificity and sensitivity limit clinical utility. Although many studies have been conducted to evaluate the clinical value of CA 19-9 in gastric cancer, there is still no general agreement.

Our meta-analysis provides important and valuable evidence for the individualized treatment for gastric cancer. Our results indicate that CA 19-9 is associated with clinicopathologic characteristics, including tumor stage, pT category, lymph node metastasis, distant metastasis, and vessel invasion, and is feasible for gastric tumor staging. Moreover, our results provide evidence that CA 19-9 can be used to predict the prognosis of gastric cancer. Similar results were obtained in the subgroup analyses stratified by sample time, cut-off value, and study quality.

Recently, several studies have reported that CA 19-9 is associated with gastric cancer metastasis and prognosis [24, 49]. Our results also obtained similar evidence. Metastatic patients had a 2.76-fold elevated level of CA 19-9 compared to patients without metastasis. Moreover, the CA 19-9 level was more frequently elevated in patients with peritoneal and hepatic metastases (2.20- and 3.13-fold, resp.). Our results also indicated that an elevated level of CA 19-9 is associated with poor prognosis in gastric cancer, including poor OS, DFS, and DSS. Marrelli et al. demonstrated that stage I patients with elevated CA 19-9 levels had a similar prognosis to stage II with normal levels, and the prognosis of stage II with elevated levels was similar to stage III with normal levels [49]. Thus, the CA 19-9 level had a very important and valuable prognostic value in patients with gastric cancer. Nevertheless, the intrinsic mechanisms by which the elevated level of CA 19-9 can result in poor prognosis are unclear. The most plausible explanation may be that CA 19-9, as a ligand of E-selectin and an intercellular adhesion molecule, plays a crucial role in intercellular adhesion of tumor cells to vascular endothelial cells and then contributes to tumor invasion and metastasis [55, 56]. Indeed, experimental studies have demonstrated that cells expressing CA 19-9 may have a greater capacity of invasion and metastasis [57–59]. In addition, Tabuchi et al. suggested that CA 19-9 may be drained by the thoracic duct of the lymphatic system via node metastasis or invasive lymphatics [60]; thus the elevated level of CA 19-9 may prompt the presence of micrometastases, which can lead to subsequent relapse/metastasis. Further studies are needed to investigate the potential mechanisms underlying the association between CA 19-9 and tumor metastasis and prognosis.

In our meta-analysis, the elevated level rate (sensitivity) of CA 19-9 ranges from 6.8% to 51.7% (median: 30.0%) and the CA 19-9 level was most frequently elevated in advanced stage tumor. Kochi et al. reported that the elevated level of CA 19-9 increased gradually with tumor stages (25.3% in stage I, 10.5% in stage II, 12.6% in stage III, and 51.6% in stage IV) [48]. Similarly, Duraker and Elk reported that the rate of advanced tumors (stages III and IV) was significantly higher in patients with elevated CA 19-9 levels than in patients with normal levels [46]. The included patients in the Tachibana et al., Jiang et al., Shimizu et al., Yamashita et al., and Kim et al. studies were mostly early stage [31, 34, 37, 40, 51], and this may be the main reason why the elevated CA 19-9 level was low (6.8%–12.6%). In contrast, other studies included advanced stage or metastatic patients, and the elevated CA 19-9 level was high (36.3%–54.1%) [10, 21, 22, 24, 28, 32, 47]. Thus, the CA 19-9 levels may reflect tumor burden and indicate tumor stage preoperatively. Future studies are needed to explore whether the clinical values of CA 19-9 levels differ in patients of different tumor stages.

However, insufficient sensitivity of CA 19-9 may limit its clinical utility; a number of studies have been conducted to resolve the problem via increasing sensitivity. Ikeda et al. reported that combination of CA 19-9 and other tumor markers could provide more useful diagnostic information for patients with gastric cancer than CA 19-9 alone [54]. Similarly, Tian et al. reported the complementary role of CA 19-9 and carcinoembryonic antigen (CEA), and the sensitivity increased with combined use [23]. Future studies are needed to explore which kinds of tumor markers can be combined with CA 19-9 to increase diagnostic accuracy obviously.

Compared with the diagnostic value, the sensitivity for predicting recurrence was relatively higher. Marrelli et al. reported that the sensitivity for recurrence of CA 19-9 was 56%, with a specificity of 74% [61]. Kim et al. reported that the sensitivity for recurrence was 68.2% in advanced gastric cancer, with a specificity of 80.0% [34]. Moreover, the combination of CA 19-9 and other tumor markers provided more useful prognostic information. Marrelli et al. reported that the sensitivity increased to 87% when CA 19-9 was combined with CEA and CA 72-4 [61]. Huang et al. obtained similar results on the prediction of recurrence [9]. Thus, CA 19-9, a low-cost and convenient test, may be useful for predicting prognosis and of use as an important indicator for high risk of recurrence.

There is no agreement on the optimal cut-off value for the prognostic value of CA 19-9. Most studies have used a cut-off value of 37 U/mL, but this cut-off value is often used for the diagnosis of gastric cancer and it is unknown whether this cut-off value is appropriate as a prognostic value. Yajima et al. determined the cut-off value of CA 19-9 as a predictor of prognosis via receiver operating characteristic analysis, and the cut-off value was ≥77 U/mL, which was approximately twice the upper limit of the normal range (37 U/mL) [20]. High-quality, well-designed multicenter studies are required to establish an optimal cut-off value for CA 19-9 and determine whether we should use different cut-off values for clinicopathologic and prognostic values.

The strength of the present meta-analysis lies in the inclusion of all relevant studies. Not only did we evaluate the clinicopathologic value of CA 19-9 in patients with gastric cancer, we also assessed the prognostic value of CA 19-9. Moreover, our results and conclusions were confirmed by in-depth subgroup analyses. Thus, our results may be valuable for diagnosis, prediction of prognosis, and individual treatment in patient with gastric cancer.

There were several limitations in the present study. First, we could not obtain detailed individual information. Thus, we could not control other potential biases. Second, there was considerable heterogeneity and heterogeneity still could not be eliminated. Third, our study did not obtain a conclusive result regarding the optimal cut-off value and could not evaluate whether chemotherapy would impact the prognostic value of CA 19-9 due to a limited number of included studies. Therefore, future studies are urgently needed to assess the cut-off value of CA 19-9 and effects of chemotherapy on CA 19-9.

## 5. Conclusions

Our meta-analysis showed that CA 19-9 may indicate clinicopathologic characteristics of gastric cancer and is associated with poor prognosis. Further high-quality and large-scale studies are required to determine whether CA 19-9 can be used for the individualized treatment for patients with gastric cancer.

## Figures and Tables

**Table 1:** Baseline characteristics and quality of the included studies.

Study	Country	Number of patients (M/F)^a^	Sample time^b^	Age mean ± SD/median (range)^c^	Rate of CA 19-9^d^	Follow-up mean ± SD/median (range)^e^	Outcome measured^f^	Study quality^g^
Huang et al. [9]	China	363 (266/97)	Preoperative	60.67 ± 11.91	67/354	Range: 41–88	DFS, OS	6
Jo et al. [10]	Korea	1178 (773/405)	Preoperative + postoperative	56 (20–88)	449/1187	28.2 (10.8–102.5)	OS	7
Ye et al. [11]	China	117 (72/45)	Preoperative	63 (28–86)	NR	38 (4–62)	DFS, OS	7
Yajima et al. [20]	Japan	23 (16/7)	Preoperative	53–86	11/23	Max: 36	OS	4
Wang et al. [21]	China	439 (319/120)	NR	NR	123/307	NR	OS	5
Turkoz et al. [22]	Turkey	176 (119/57)	NR	57.8 ± 12.9	56/176	27.5 (1–56.5)	OS	6
Tian et al. [23]	China	181 (121/60)	Preoperative	Mean: 59.5	31/181	Max: 60	OS	6
Mohri et al. [24]	Japan	123 (38/85)	NR	66 (18–94)	47/123	Median: 9.3	OS	6
Kanda et al. [25]	Japan	238 (179/59)	Preoperative	65.3 ± 11.7	44/238	Max: 60	DSS	6
Choi et al. [29]	Korea	488 (333/155)	Preoperative	Range: 27–85	163/488	Max: 44	DFS, OS	6
Mittal et al. [26]	Nepal	40 (27/13)	Preoperative	NR	16/40	Max: 60	OS	6
Li et al. [27]	China	1501 (1073/428)	Preoperative	58.96 ± 11.93	284/1501	Max: 60	OS	6
Kwon et al. [28]	Korea	102 (41/61)	Postoperative	NR	79/102	Max: 144	OS	6
Liu et al. [30]	China	273 (192/81)	Preoperative	56 ± 12	87/273	Median: 61.2	OS	6
Jiang et al. [31]	Japan	1710 (1157/553)	Preoperative	NR	151/1557	43 (1–123)	OS	7
Emoto et al. [32]	Japan	102 (54/48)	NR	57 (28–79)	37/102	Max: 48	OS	5
Schauer et al. [33]	Germany	120 (60/60)	Preoperative	Mean: 57.7; range: 28–83	36/120	38 (2–120)	OS	6
Kim et al. [34]	Korea	167 (108/59)	Postoperative	NR	21/167	59.6 ± 12.7; 60.7 (9.8–84.8)	DFS	6
Dilege et al. [35]	Turkey	75 (47/28)	Preoperative	NR	25/75	24 (6–74)	OS	6
Zhang et al. [36]	China	166 (116/50)	Preoperative	58 (24–85)	27/166	Median: 18	OS, DFS	5
Yamashita et al. [37]	Japan	382 (263/119)	Preoperative	Mean: 59; range: 21–86	39/382	Mean: 45; range: 0–60	DSS	7
Ucar et al. [38]	Turkey	95 (63/32)	Preoperative	58 ± 10	39/95	Median: 18	OS	5
Yamashita et al. [39]	Japan	128 (86/42)	Preoperative	Mean: 59; range: 21–86	24/128	Max: 60	DSS	7
Shimizu et al. [40]	Japan	40 (28/12)	Preoperative	Mean: 60.6; range: 28–86	4/40	31.7 (1–48)	OS	6
Louhimo et al. [41]	Finland	146 (73/73)	Preoperative	63.8 (31.6–88.4)	45/146	13.4 (0–166.8)	DSS	7
Nakagoe et al. [42]	Japan	218 (144/74)	Preoperative	NR	45/218	62.3 (1.3–117.3)	DSS	7
Lai et al. [43]	Taiwan	192 (120/72)	Preoperative	NR	31/192	Mean: 58; range: 6–182	DFS	6
Duraker and Elk [46]	Turkey	168 (116/52)	Preoperative	NR	53/168	Max: 50	OS	6
Ishigami et al. [44]	Japan	549 (386/168)	Preoperative	NR	109/549	42 (12–76)	OS	5
Gaspar et al. [45]	Spain	82 (55/27)	Preoperative	63 ± 12	27/82	Median: 16	OS	5
Ychou et al. [47]	France	52 (40/12)	Preoperative	62 ± 12.3	26/52	Max: 48	OS	5
Kochi et al. [48]	Japan	435 (317/118)	preoperative + postoperative	61.9 (20–90)	95/435	Max: 100	DFS	6
Marrelli et al. [49]	Italy	153 (95/58)	Preoperative	69 ± 10	53/153	74 ± 10	OS	7
Tocchi et al. [50]	Italy	59 (40/19)	Preoperative	Mean: 64.3; range: 31–84	23/59	Max: 60	OS	7
Tachibana et al. [51]	Japan	196 (136/60)	Preoperative	NR	13/192	NR	DSS	6
Nakajima et al. [52]	Japan	110 (75/35)	Preoperative	63 ± 10	29/105	Max: 36	OS	5
Kodera et al. [53]	Japan	663 (432/231)	Preoperative	NR	106/663	Max: 50	OS	5
Ikeda et al. [54]	Japan	158 (99/59)	Preoperative	NR	42/158	Max: 36	OS	5

*Note.* CA 19-9: carbohydrate antigen 19-9; DFS: disease-free survival; DSS: disease-specific survival; max: the maximum of follow-up time; M/F: male/female; NR: not reported; OS: overall survival; SD: standard deviation.

^a^The total number of patients in each included study, with displaying them as male/female.

^b^The sample time was defined according to the time of operation.

^c^The mean age of patients with corresponding SD or median age with corresponding range.

^d^The rate of patients with high level of CA 19-9.

^e^The mean follow-up period with corresponding SD or median follow-up period with corresponding range.

^f^The outcomes assessed (DFS, DSS, or/and OS) were presented in each included study.

^g^The quality of the included studies was assessed with the nine-star Newcastle-Ottawa Scale criteria.

**Table 2:** Detailed results of subgroup analyses.

	Sample time^a^		Cut-off^b^		Study quality^c^	
	Any	Preoperative	37	Other than 37	NOS ≥6	NOS <6
Stages III/IV versus I/II (OR^d^)	3.36 [2.34, 4.84]; *I* ^2^ = 71.6%^e^	3.50 [2.34, 5.23]; *I* ^2^ = 72.9%	3.17 [2.04, 4.94]; *I* ^2^ = 78.5%	3.81 [2.40, 6.06]; *I* ^2^ = 39.8%	2.75 [1.85, 4.10]; *I* ^2^ = 64.8%	4.56 [2.77, 7.50]; *I* ^2^ = 48.8%
pT: T3/T4 versus T1/T2 (OR)	2.40 [1.60, 3.59]; *I* ^2^ = 62.8%	2.59 [1.65, 4.07]; *I* ^2^ = 62.7%	2.51 [1.58, 4.00]; *I* ^2^ = 70.2%	2.11 [1.15, 3.88]; *I* ^2^ = 44.4%	2.41 [1.48, 3.94]; *I* ^2^ = 68.6%	2.62 [1.69, 4.07]; *I* ^2^ = 49.2%
Lymph node-positive versus negative (OR)	2.91 [2.21, 3.84]; *I* ^2^ = 55.3%	3.15 [2.36, 4.12]; *I* ^2^ = 54.3%	3.07 [2.14, 4.41]; *I* ^2^ = 62.4%	2.29 [1.77, 2.96]; *I* ^2^ = 4.2%	2.47 [1.81, 3.36]; *I* ^2^ = 40.6%	3.62 [2.36, 5.56]; *I* ^2^ = 50.8%
Metastasis-positive versus negative (OR)	2.76 [1.12, 6.82]; *I* ^2^ = 14.9%	2.76 [1.12, 6.82]; *I* ^2^ = 14.9%	/	/	/	/
Peritoneal dissemination positive versus negative (OR)	2.20 [1.25, 3.90]; *I* ^2^ = 85.3%	2.55 [1.35, 4.83]; *I* ^2^ = 74.7%	2.18 [0.95, 4.99]; *I* ^2^ = 90.0%	2.23 [1.03, 4.83]; *I* ^2^ = 62.4%	1.43 [0.68, 3.05]; *I* ^2^ = 77.1%	2.98 [1.59, 5.59]; *I* ^2^ = 71.3%
Hepatic metastasis-positive versus negative (OR)	3.13 [1.50, 6.55]; *I* ^2^ = 76.9%	3.82 [1.98, 7.36]; *I* ^2^ = 56.2%	3.27 [1.40, 7.65]; *I* ^2^ = 70.4%	3.13 [0.83, 11.82]; *I* ^2^ = 71.5%	1.71 [0.34, 8.68]; *I* ^2^ = 76.6%	4.01 [1.61, 9.99]; *I* ^2^ = 80.3%
Lauren type: diffuse versus intestinal (OR)	0.89 [0.62, 1.27]; *I* ^2^ = 0.0%	0.89 [0.62, 1.27]; *I* ^2^ = 0.0%	0.78 [0.47, 1.27]; *I* ^2^ = 0.0%	1.04 [0.62, 1.76]; *I* ^2^ = 0.0%	0.83 [0.53, 1.29]; *I* ^2^ = 0.0%	1.02 [0.55, 1.90]; *I* ^2^ = 0.0%
Differentiated: poor versus well + moderate (OR)	0.87 [0.72, 1.04]; *I* ^2^ = 3.4%	0.85 [0.70, 1.03]; *I* ^2^ = 17.3%	0.87 [0.72, 1.06]; *I* ^2^ = 28.5%	0.86 [0.49, 1.50]; *I* ^2^ = 0.0%	0.74 [0.57, 0.98]; *I* ^2^ = 25.5%	0.99 [0.77, 1.27]; *I* ^2^ = 0.0%
Vessel invasion-positive versus negative (OR)	1.66 [1.11, 2.48]; *I* ^2^ = 0.0%	2.24 [1.09, 4.60]; *I* ^2^ = 0.0%	/	1.99 [1.11, 3.55]; *I* ^2^ = 0.0%	1.68 [1.03, 2.73]; *I* ^2^ = 40.4%	1.64 [0.81, 3.30]; *I* ^2^ = 0.0%
Lymphatic invasion-positive versus negative (OR)	1.44 [0.91, 2.28]; *I* ^2^ = 0.0%	1.20 [0.57, 2.51]; *I* ^2^ = 24.2%	1.36 [0.82, 2.25]; *I* ^2^ = 30.8%	/	1.36 [0.82, 2.25]; *I* ^2^ = 30.8%	/
OS (HR^f^)	1.83 [1.56, 2.15]; *I* ^2^ = 75.8%	1.87 [1.52, 2.30]; *I* ^2^ = 76.3%	1.87 [1.53, 2.30]; *I* ^2^ = 78.5%	1.78 [1.30, 2.43]; *I* ^2^ = 71.8%	1.72 [1.47, 2.01]; *I* ^2^ = 69.6%	1.99 [1.31, 3.04]; *I* ^2^ = 78.6%
DFS (HR)	1.85 [1.16, 2.95]; *I* ^2^ = 60.2%	1.96 [1.00, 3.85]; *I* ^2^ = 72.9%	1.55 [1.15, 2.08]; *I* ^2^ = 0.0%	3.15 [0.68, 14.63]; *I* ^2^ = 85.6%	1.54 [1.16, 2.03]; *I* ^2^ = 0.0%	/
DSS (HR)	1.33 [1.10, 1.60]; *I* ^2^ = 17.5%	1.33 [1.10, 1.60]; *I* ^2^ = 17.5%	1.36 [1.12, 1.65]; *I* ^2^ = 17.4%	/	1.33 [1.10, 1.60]; *I* ^2^ = 17.5%	/

*Note.* DFS: disease-free survival; DSS: disease-specific survival; HR: hazard ratio; NOS: Newcastle-Ottawa Scale; OR: odds ratio; OS: overall survival; “/” symbol: no results due to insufficient studies.

^a^Subgroup analysis was stratified by sample time. “Any” means including preoperative and postoperative sample time. “Preoperative” means including preoperative sample time only.

^b^Subgroup analysis was stratified by cut-off value: 37 U/mL and other than 37 U/mL.

^c^Subgroup analysis was stratified by study quality: NOS ≥6 and NOS <6.

^d^OR was used as effect measure to assess the result of carbohydrate antigen 19-9 detection in different clinicopathologic characteristics.

^e^The result was presented together with its 95% confidence intervals (in square brackets). Furthermore, *I* ^2^ statistics in the pooled analyses were presented.

^f^HR was used as effect measure to assess the relationship between carbohydrate antigen 19-9 and prognosis.
